# Comparison of short-segment vs. long-segment fixation in posterior osteotomy for kyphotic deformity: effects on postoperative alignment and complications

**DOI:** 10.3389/fsurg.2026.1771214

**Published:** 2026-02-12

**Authors:** Yulong Zhao, Qian Yuan, Na Zhang, Lin Chen, Shiduo Zhang, Qiang Li

**Affiliations:** Department of Orthopaedics (I), North China Medical Health Group XingTai General Hospital, Xingtai, Hebei, China

**Keywords:** kyphotic deformity, long-segment fixation, osteotomy, posterior osteotomy, short-segment fixation

## Abstract

**Background:**

Posterior osteotomy is an effective treatment for severe kyphosis; however, postoperative complications, particularly early radiographic proximal junctional kyphosis (PJK), may compromise outcomes. Evidence regarding factors influencing postoperative efficacy and complication risk remains limited.

**Objective:**

To compare the clinical efficacy of long-segment vs. short-segment fixation in posterior osteotomy for kyphosis and identify factors affecting outcomes and complications.

**Methods:**

This retrospective controlled study included 150 patients undergoing posterior thoracolumbar osteotomy with fusion and internal fixation between June 2019 and June 2023. Patients were grouped by fixation length: long-segment (group L, ≥5 segments) and short-segment (group S, ≤4 segments). Propensity score matching balanced baseline characteristics, yielding 50 patients per group. Radiographic parameters (Cobb angle, sagittal vertical axis), perioperative data, and 12-month complications were recorded. Pain and function were assessed using VAS and ODI. Multivariate logistic regression identified independent risk factors for correction loss and early radiographic PJK.

**Results:**

Both groups showed significant postoperative improvement in Cobb angle and sagittal alignment (*P* < 0.05). Group L achieved a higher correction rate, lower Cobb angle loss, better VAS and ODI scores, and fewer early radiographic PJK and fixation-related complications (all *P* < 0.05). Group S had shorter operative time and less blood loss (*P* < 0.05), with no difference in other complications. Short-segment fixation was independent risk factors for correction loss and early radiographic PJK.

**Conclusion:**

When correcting spinal kyphosis via posterior osteotomy, long-segment fixation better maintains correction and reduces complications like early radiographic PJK, while short-segment fixation shortens surgery time but increases risks of correction loss and early radiographic PJK.

## 1 Introduction

Kyphotic deformity is an abnormal force line across the coronal and sagittal planes of the spine caused by congenital dysplasia, trauma, tuberculosis, ankylosing spondylitis and other reasons. It manifests as abnormal bending of the spine backward, which not only exerts a serious impact on patients’ physical appearance but may also induce multiple problems such as low back discomfort, nerve compression, cardiopulmonary dysfunction due to the disorder of spinal mechanical structure, and seriously reduces the quality of life of patients (1). The disease can be caused by congenital vertebral dysplasia (such as hemivertebrae, wedge-shaped vertebrae), spinal tuberculosis, ankylosing spondylitis, traumatic fracture or osteoporotic compression fracture and other etiologies (2). With the development of modern imaging and spinal surgery technology, posterior osteotomy incorporating bone graft fusion and internal fixation has become the mainstream method for the treatment of moderate to severe kyphosis. Its core goal is to correct structural deformities by osteotomy, promote bone fusion by bone graft, maintain the orthopedic effect and rebuild spinal stability by internal fixation system (3).

Within this surgical system, the selection of fixation segments—specifically long segment fixation (LSF) or short segment fixation (SSF)—stands as one of the critical factors influencing surgical outcomes. LSF usually spans multiple adjacent normal vertebral bodies superior and inferior to the deformity area, and theoretically can provide stronger biomechanical stability, effectively reduce focal stress accumulation at the junctional zone after operation, which helps decrease the risk of orthopedic loss and early radiographic proximal junctional kyphosis (PJK) (4). However, the cost is to sacrifice more spinal motion segments, increase surgical duration, intraoperative hemorrhage, and soft tissue parameters dissection range, which may cause the acceleration of adjacent segment degeneration (ASD) (5). In contrast, SSF only fixes the core segment of the lesion and its directly adjacent vertebral body, featuring the advantages of reduced surgical invasion, shorter procedural time, and superior retention of motor function, and is in line with the concept of minimally invasive spine surgery (6). However, its mechanical strength is relatively limited. When dealing with patients with severe deformities or osteoporosis, there are risks of internal fixation failure, reduction loss and long-term correction loss (7). Understanding the curative effect of long and short segment fixation, postoperative adverse effects and risk factors of kyphosis recurrence can improve our medical level, better avoid risks and reduce postoperative complications. At the same time, the hospital stay of patients was shortened, the treatment cost was reduced, and better postoperative effect was obtained.

In current clinical practice, there is still a lack of unified standards for the selection of LSF and SSF, which is controversial. Several retrospective studies have tried to compare the efficacy of the two, but most of them did not fully control the confounding bias, which affected the reliability of the results. For example, retrospective cohort analysis of 127 adult cases of degenerative scoliosis by Jiang et al. found that although LSF has more advantages in correcting spinal imbalance, its perioperative complication rate is significantly higher than that of SSF, suggesting that the benefits and risks need to be weighed (8). Similarly, Zhang et al.'s. study also pointed out that implementation of long segment internal fixation represents an independent risk factor contributing to medical complications following surgery in elderly patients with ADS (9). These studies highlight the importance of controlling for baseline differences when comparing between groups.

In order to overcome the limitations of traditional comparison methods, probability score matching (PSM), as an effective statistical tool, is widely utilized in spinal surgical studies to minimize selection bias (10). Through PSM to balance the distribution of key covariates such as age, sex, basic disease, preoperative deformity degree between the two groups, the comparison between SSF and L group can be closer to the level of randomized controlled trials, so as to improve the reliability of the conclusion. In addition, combined with multivariate linear regression or logistic regression analysis, it can further identify independent predictors of adverse outcomes such as postoperative orthopedic loss, internal fixation failure, and early radiographic PJK, such as low Bone Mineral Density (BMD), severe preoperative imbalance, improper selection of upper fixed vertebra (UIV), and no use of transverse devices (11, 12).

In view of this, this study used a retrospective clinical control study method to compare and analyze the perioperative indicators, imaging parameters, functional recovery and complications of the two groups of patients with kyphosis who were undergoing LSF or SSF, accordingly. The clinical application value of the two fixation methods was objectively evaluated, and the linear regression analysis was utilized to examine the influencing factors, thereby furnishing a scientific foundation for the individualized selection of clinical fixation strategies and the prevention and control of related risks.

## 2 Methods

### 2.1 Study subjects

**Figure 1:**
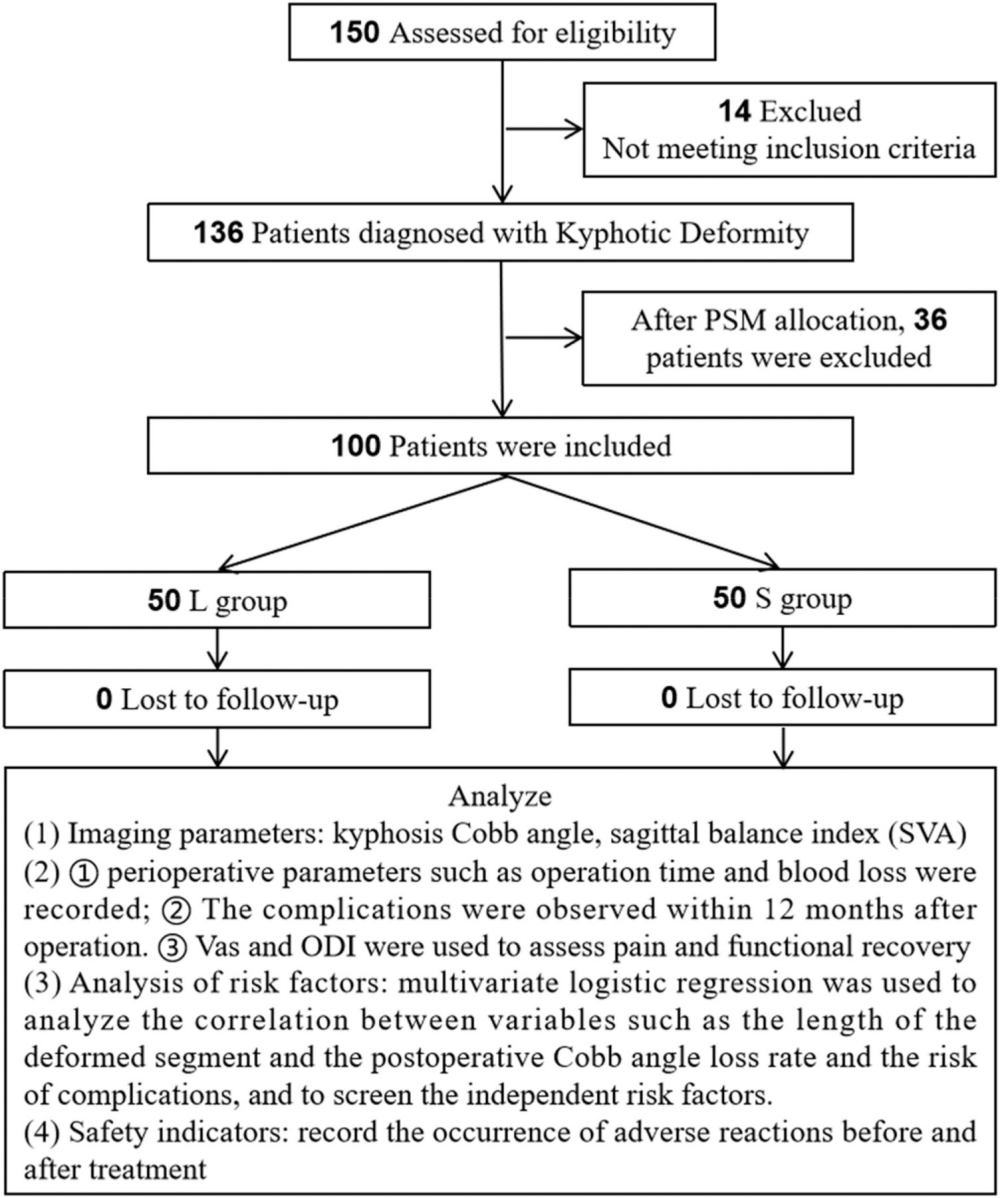
Research flowchart.

This study is a retrospective clinical study. It studies the medical records of patients with kyphosis who underwent posterior thoracolumbar osteotomy, orthopedic bone graft fusion and internal fixation in Xingtai General Hospital of North China Medical and health group from June 2019 to June 2023. Initially, 150 patients with kyphosis who underwent posterior osteotomy, orthopedic bone graft fusion and internal fixation were selected. After screening against the inclusion and exclusion criteria, 14 patients were excluded. For reducing selection bias, 1:1 PSM was performed based on relevant baseline data (gender, age, etc.), with the matching caliper width set to 0.05. Finally, 100 patients were included, 50 in group L and 50 in group S. The perioperative indicators, imaging parameters, comparative assessment of functional recovery and complications was performed between the two groups. At the same time, the linear regression model was used to screen the risk factors, objectively evaluate the clinical efficacy differences of the two fixation methods and analyze the influencing factors, thereby supporting evidence-based decision-making in the individualized choice of clinical fixation strategies and the proactive mitigation of potential risks. The flow chart is shown in Figure 1.

#### 2.1.1 PSM methodology

To minimize selection bias and enhance comparability between the long-segment (Group L) and short-segment (Group S) fixation groups, 1:1 PSM was performed using SPSS 25.0. The propensity score was estimated via a logistic regression model that included the following baseline covariates: age, sex, body mass index (BMI), preoperative Cobb angle, BMD T-score, length of the deformity segment, duration of disease, and location of the deformity segment (thoracic, thoracolumbar, or lumbar). These variables were selected based on their potential influence on both the choice of fixation strategy and surgical outcomes.

Matching was performed using the nearest-neighbor algorithm with a caliper width set to 0.05 of the standard deviation of the logit of the propensity score, without replacement. Balance of covariates between the two groups before and after matching was assessed using standardized mean differences (SMD), with an SMD < 0.10 considered indicative of good balance.

Table 1A After PSM, all included covariates achieved adequate balance (SMD < 0.10), confirming the effectiveness of the matching procedure in reducing baseline differences between Group L and Group S. The sample size after matching was 50 patients per group (total *n* = 100), which satisfied the statistical power requirement for subsequent analyses.

**Table 1A:** Standardized mean differences of baseline covariates before and after PSM.

Covariate	Before matching (SMD)	After matching (SMD)
Age	0.12	0.03
Sex (female)	0.08	0.02
BMI	0.05	0.04
Preoperative Cobb angle	0.09	0.05
BMD T-score	0.07	0.03
Deformity segment length	0.1	0.06
Duration of disease	0.15	0.07
Deformity location	0.06	0.04

SMD, standardized mean difference.

### 2.2 Inclusion criteria

(1) Kyphotic deformity was confirmed by imaging examination, with Cobb angle ≥40° (13); (2) Posterior osteotomy, orthopedic bone graft, fusion and internal fixation were performed; (3) The fixation methods were simple LSF (≥5 segments) or SSF (≤4 segments), without mixed fixation; (4) Patients with thoracolumbar fracture and kyphosis before operation had or did not have lower limb neurological symptoms; (5) The clinical data were complete, including preoperative, immediate postoperative and 12-month postoperative imaging data, operation records, follow-up records, BMD test results, etc; (6) Follow up time ≥12 months; (7) The patient has clear consciousness and can cooperate to complete the functional score and follow-up evaluation.

### 2.3 Exclusion criteria

(1) Kyphosis caused by congenital spinal deformity, spinal tumor, tuberculosis, infection and fracture (14); (2) Previous history of spinal surgery (13); (3) Complicated with severe heart, liver, kidney and other organ dysfunction, unable to tolerate surgery; (4) Combined with nervous system disease, affecting postoperative functional evaluation; (5) Allergic to bone graft material.

### 2.4 Ethics statement

This study is a retrospective human clinical study involving human subjects. This study protocol was approved by the ethics committee of the hospital. Both data acquisition and retrospective analytical procedures adhered to the requirements specified in the National Ethical Review Measures for Biomedical Research Involving Humans and the Declaration of Helsinki (15). The study used anonymous retrospective medical record analysis without direct contact with subjects. All identifiable personal information has been de identified to ensure data confidentiality.

### 2.5 Sample size calculation

This study is a retrospective clinical controlled trial. Patients were assigned to group L or group S according to the treatment method. The study used independent sample *t*-test for data analysis and G-Power software for sample size calculation (16). With “Cobb angle loss” as the primary endpoint, based on previous studies (17), the selected effect size was 0.8, the significance level (α) was 0.05 (two-sided), and the statistical power (1-β) was 0.95. These parameters resulted in the need for 35 participants in each group, a total of 70. Finally, a total of 100 participants were included in this study, which exceeded the target sample size and met the statistical requirements of the study.

### 2.6 Therapeutic method

The categorization of LSF as ≥5 fused levels and SSF as ≤4 levels was based on established clinical literature (17). This extended construct shifts the high-stress junction away from the osteotomy site to more proximal, anatomically normal vertebrae, thereby reducing the risk of PJK and correction loss.

The same group of doctors performed posterior osteotomy, orthopedic bone graft fusion and internal fixation in the two groups. The surgical approach, osteotomy method and bone graft material were the same. In group S, SSF was used: the fixed segment included the deformed core segment and one normal vertebral body at the upper and lower levels, and the number of fixed segments was ≤4. The pedicle screw internal fixation system with appropriate length was selected, and the bone graft material was autologous iliac bone or allogeneic bone. group L was fixed with long segments: the fixed segments included the deformed core segment and more than 2 normal vertebral bodies at the upper and lower levels, and the number of fixed segments was ≥5. The internal fixation system and bone graft material were the same as those in group S. The patients in both groups were given conventional treatments such as anti-infection, pain relief, nutritional support, and early rehabilitation training under the guidance of doctors (18).

### 2.7 Observation index

#### 2.7.1 Imaging parameters

(1) The Cobb angle of kyphosis (19) was collected to quantify the severity of kyphosis. Degree of Cobb angle loss = Cobb angle 12 months after surgery—Cobb angle immediately after surgery.

(2) Sagittal balance index (SVA) (19) of patients was collected to evaluate the core global index of the overall sagittal alignment of the spinal force line. SVA<5 cm was considered as good sagittal balance; Notably, the sagittal plane assessment in this study was limited to SVA, and pelvic parameters [including pelvic incidence [PI], pelvic tilt [PT], lumbar lordosis [LL], and PI–LL mismatch] were not included due to the focus on global sagittal alignment restoration directly related to kyphotic deformity correction.

(3) Poor alignment rate of vertebral body: the proportion of patients with vertebral body displacement and angulation ≥ 3° in postoperative imaging examination.

#### 2.7.2 Secondary indicators

(1) Perioperative parameters such as operation time and blood loss were recorded (18).

(2) The incidence of complications such as early radiographic PJK (New kyphosis at the upper and lower ends of the fixed segment, Cobb angle ≥ 10°, identified on 12-month follow-up imaging), loosening/fracture of internal fixation, bone graft non fusion (no callus growth or pseudoarthrosis in the bone graft area 12 months after operation), nerve injury (new limb numbness, weakness and other neurological symptoms after operation, confirmed by electromyography) and other complications were observed.

(3) Visual analogue scale (VAS) and Oswestry disability index (ODI) were used to evaluate pain and functional recovery (20).

#### 2.7.3 Relevant indicators of risk factor analysis

Multivariate logistic regression was used to analyze the correlation between variables such as the length of the malformed segment and the postoperative Cobb angle loss rate and the risk of early radiographic PJK, and to screen the independent risk factors.

Independent variables included: (1) demographic characteristics: age, gender (dichotomous variables: male = 0, female = 1) BMI; (2) Disease related indicators: preoperative Cobb angle (<60° = 0, ≥ 60° = 1), BMD T value (≥ −2.5 = 1, <−2.5 = 0); (3) Operation related indicators: fixation method (long segment = 1, short segment = 0), operation time, intraoperative blood loss (<800 mL = 0, ≥800 mL = 1); ④ Comorbidities (yes = 1, no = 0): hypertension, diabetes, osteoporosis.

Dependent variables included: (1) postoperative Cobb angle loss rate; (2) Occurrence of early radiographic PJK (occurrence = 1, non occurrence = 0).

#### 2.7.4 Safety index

Adverse reaction records: medical electronic sphygmomanometer (HEM-907, Omron) was used for blood pressure monitoring; Blood routine examination was performed using a fully automatic blood cell analyzer (DXH 800, Beckman Coulter); Electrocardiography was performed using an electrocardiograph (IMEC 12, Mindray). The medical staff closely observed the patient's physical condition and reaction during the treatment, and collected adverse reaction information through detailed medical records and patient self-report. MedDRA (21) was used as a standardized tool to ensure that the description and grading of adverse reactions were more accurate and standardized.

### 2.8 Statistical analysis

SPSS 25.0 statistical software was used for data analysis. For age and other measurement data, the Kolmogorov Smirnov test method is used to test the normality. If it conforms to the normal distribution, it is expressed by the mean ± standard deviation. The independent sample *t*-test is selected for comparison between groups, and the paired t test is used for the same group before and after treatment. For counting data such as gender, it is expressed by [*n* (%)], and chi-square (*χ*²) test is used for comparison between groups. The difference was considered statistically significant when *P* < 0.05.

## 3 Results

### 3.1 Comparison of patient baseline data

Table 1B presents the baseline characteristics of patients in group L (*n* = 50) and group S (*n* = 50) after PSM allocation. There was no significant difference between the two groups in gender, age, BMI, Cobb angle, BMD T value, length of deformity segment, duration of disease and location of deformity segment (*P* > 0.05), indicating that the two groups had balanced baseline and good comparability.

**Table 1B:** Baseline characteristics [mean ± SD, *n* (%)].

Variables	L group	S group	χ²/t	*P*
*n*	50	50	–	–
Gender				
Male	22 (44.0)	21 (42.0)	0.041	0.840
Female	28 (56.0)	29 (58.0)		
Age (years)	60.6 ± 2.0	59.7 ± 2.1	1.248	0.215
Age > 60	28 (56.0)	20 (40.0)	2.564	0.109
BMI	24.6 ± 2.8	24.8 ± 2.4	0.558	0.578
BMI > 25	21 (42.0)	23 (46.0)	0.162	0.687
Cobbs angle (°)	45.3 ± 3.6	45.2 ± 2.2	0.172	0.864
Cobbs angle ≥ 45°	23 (46.0)	31 (62.0)	2.577	0.109
BMD T value	−2.4 ± 0.4	−2.4 ± 0.3	1.044	0.299
BMD T value < −2.5	15 (30.0)	22 (44.0)	2.102	0.147
Deformity segment length (*n*)	3.0 ± 1.1	2.8 ± 1.2	0.337	0.737
Deformity segment length < 3	13 (26.0)	14 (28.0)	0.051	0.822
Duration (years)	9.2 ± 3.0	10.2 ± 3.6	1.615	0.110
Duration ≥ 10	22 (44.0)	28 (56.0)	1.440	0.230
Deformity segment location				
Thoracic segment	24 (48.0)	23 (46.0)	0.046	0.977
Thoracolumbar segment	20 (40.0)	21 (42.0)		
Lumbar segment	6 (12.0)	6 (12.0)		

BMI, body mass index.

### 3.2 Comparison of imaging parameters between the two groups

Table 2 presents the following key findings: No statistically significant difference was observed in the kyphotic Cobb angle or SVA between the two groups preoperatively (*P* > 0.05). Immediately postoperatively, both groups exhibited significant improvements in Cobb angle and SVA (*P* < 0.05), with the long-segment fixation group (group L) demonstrating a significantly higher Cobb angle correction rate compared to the short-segment fixation group (group S) (*P* < 0.001). At the 12-month postoperative follow-up, group L showed a significantly lower degree of Cobb angle loss than group S (*P* = 0.001), and its excellent-and-good rate of SVA (92%) was notably higher than that of group S (72%) (*P* < 0.001). Additionally, the incidence of poor vertebral alignment in group L (4%) was significantly lower than in group S (12%) (*P* = 0.037).

**Table 2:** Comparison of imaging parameters between two groups.

Variables	Time	L group (*n* = 50)	S group (*n* = 50)	χ²/t	*P*
Cobb angle (°)	Before treatment	45.3 ± 3.6	45.2 ± 2.2	0.172	0.864
	After operation	7.9 ± 3.0*	11.0 ± 2.9*	5.243	<0.001
	After 12 months	10.3 ± 2.8	15.3 ± 1.9	10.213	<0.001
	Loss	2.4 ± 2.4	4.3 ± 2.6	3.763	<0.001
SVA (cm)	Before treatment	9.6 ± 2.3	8.7 ± 2.5	1.796	0.076
	After operation	2.3 ± 0.8*	3.5 ± 0.9*	6.947	<0.001
	After 12 months	3.3 ± 0.8	4.9 ± 1.0	9.048	<0.001
	Excellent rate	92.0%	72.0%	13.550	<0.001
Vertebral malalignment rate		4.0%	12.0%	4.348	0.037

Compared with that before treatment in the same group.

* *P* < 0.05. The same below.

### 3.3 Comparison of perioperative indicators between the two groups

Table 3 reveals that the group S had significantly shorter operative time and less intraoperative blood loss compared to the group L (both *P* < 0.001), while no statistically significant difference was noted in the length of hospital stay between the two groups (*P* = 0.393).

**Table 3:** Comparison of perioperative indicators between two groups (mean ± SD).

Variables	L group (*n* = 50)	S group (*n* = 50)	t	*P*
Operation time (min)	215.6 ± 32.4	158.4 ± 25.6	9.779	<0.001
Intraoperative blood loss (mL)	985.6 ± 215.8	625.3 ± 156.7	9.551	<0.001
Length of hospital stay (days)	13.8 ± 3.0	14.3 ± 3.2	0.858	0.393

### 3.4 Comparison of complications between the two groups

Table 4 reveals that early radiographic PJK and internal fixation loosening/fracture occurred significantly less frequently in group L than in group S (*P* < 0.05). The incidence of bone graft nonunion and nerve injury did not differ significantly between the two groups (*P* > 0.05), and the overall complication rate was significantly lower in group L than in group S (*P* = 0.001).

**Table 4:** Comparison of complication rates between two groups [*n* (%)].

Variables	L group (*n* = 50)	S group (*n* = 50)	χ²	*P*
Early radiographic PJK	4 (8.0)	13 (26.0)	5.741	0.017
Internal fixation loosening/fracture	1 (2.0)	7 (14.0)	4.891	0.027
Nonunion of bone graft	2 (4.0)	3 (6.0)	0.211	0.646
Nerve injury	1 (2.0)	1 (2.0)	0	1.000
Total complications	8 (16.0)	24 (48.0)	11.765	0.001

PJK, proximal junctional kyphosis.

### 3.5 Comparison of functional assessment between the two groups

As presented in Table 5, preoperative VAS scores and ODI indices did not differ significantly between the two groups (*P* > 0.05). Twelve months after surgery, both groups had significantly reduced VAS and ODI values relative to their preoperative measurements (*P* < 0.05), and group L had notably lower VAS and ODI scores than group S (*P* < 0.001).

**Table 5:** Comparison of VAS scores and ODI indices between two groups (mean ± SD).

Variables	Time	L group (*n* = 50)	S group (*n* = 50)	t	*P*
VAS	Before treatment	7.5 ± 2.2	7.5 ± 2.0	0.188	0.852
	After 12 months	1.9 ± 0.6	2.7 ± 1.1	4.635	<0.001
ODI	Before treatment	42.3 ± 6.7	43.6 ± 5.7	1.049	0.297
	After 12 months	18.3 ± 5.2	23.8 ± 4.0	5.873	<0.001

### 3.6 Analysis of risk factors for postoperative adverse outcomes

#### 3.6.1 Multivariate linear regression analysis of postoperative cobb angle loss rate

Table 6: Using Cobb angle loss rate as the dependent variable, baseline features such as fixation method and gender were included in the multiple linear regression model. The results showed that SSF (*β* = 1.816, *P* = 0.001) was an independent risk factor for increased Cobb angle loss rate after surgery (R^2^ = 0.197, F = 2.49, *P* = 0.015).

**Table 6:** Multivariate linear regression analysis of risk factors for postoperative cobb angle loss rate.

Variables	*β*	SE	t	*P*	95% CI for β		VIF
					Lower limits	Upper limits	
Constant	3.582	0.922	3.886	<0.001	1.751	5.413	–
Short-segment	1.816	0.529	3.435	0.001	0.766	2.867	1.115
Female	−0.341	0.511	−0.668	0.506	−1.356	0.674	1.02
Age > 60	−1.061	0.56	−1.896	0.061	−2.173	0.051	1.247
BMI > 25	−0.415	0.625	−0.664	0.508	−1.656	0.826	1.533
Preoperative Cobb angle ≥ 45°	−0.222	0.515	−0.431	0.668	−1.244	0.801	1.05
Bone mineral density T value < −2.5	−0.431	0.699	−0.617	0.539	−1.819	0.957	1.815
Deformity segment length >3	0.782	0.673	1.163	0.248	−0.554	2.119	1.424
Duration >10	−0.044	0.508	−0.087	0.931	−1.053	0.965	1.028
Lumbar segment	−0.111	0.376	−0.296	0.768	−0.857	0.635	1.021

R² = 0.197, F = 2.49, *P* = 0.015.

#### 3.6.2 Binary logistic regression analysis of early radiographic PJK

Table 7: Using early radiographic PJK as the dependent variable, baseline features such as fixation method and gender were included in the binary logistic regression model. The results showed that SSF (OR = 5.552, *P* = 0.012) was an independent risk factor for the occurrence of early radiographic PJK (*χ*² = 13.310, df = 10, *P* = 0.207, Nagelkerke R² = 0.208), as shown in Table 7.

**Table 7:** Binary logistic regression analysis of risk factors for early radiographic PJK.

Variables	B	SE	χ²	*P*	OR	95% CI for OR	
						Lower limits	Upper limits
Short-segment	1.714	0.682	6.321	0.012	5.552	1.459	21.128
Female	−0.31	0.608	0.26	0.61	0.733	0.223	2.416
Age > 60	0.301	0.602	0.25	0.617	1.351	0.416	4.393
BMI > 25	−0.739	0.686	1.161	0.281	0.478	0.125	1.831
Preoperative Cobb angle ≥45°	−0.787	0.621	1.607	0.205	0.455	0.135	1.537
Bone mineral density T value < −2.5	−0.626	0.728	0.738	0.39	0.535	0.128	2.230
Deformity segment length > 3	0.375	0.709	0.281	0.596	1.456	0.363	5.837
Duration > 10	0.759	0.606	1.569	0.21	2.137	0.651	7.012
Thoracolumbar segment	0.385	0.629	0.375	0.54	1.47	0.428	5.043
Lumbar segment	−0.62	1.197	0.268	0.605	0.538	0.052	5.617
Constant	−2.386	0.97	6.051	0.014	0.092	–	–

χ²= 8.423, df = 7, *P* = 0.297, Nagelkerke R² = 0.643.

### 3.7 Comparison of adverse reaction rate during treatment

Before and after treatment, the blood routine, liver and kidney function, coagulation function and other laboratory indicators of the two groups were not significantly abnormal, and the vital signs were stable. See Table 8 for details. Symptomatic management led to the resolution of adverse reactions in both groups, with no severe adverse events reported. Statistical analysis revealed no statistically significant difference in the incidence of adverse reactions between the two groups (*χ*² = 0.154, *P* = 0.695).

**Table 8:** Comparison of adverse reaction rates during treatment [*n* (%)].

Group	L group	S group	χ²	*P*
*n*	50	50		
Lower extremity deep venous thrombosis	1 (2.0)	0		
Incision infection	2 (4.0)	2 (4.0)		
Postoperative low fever	1 (2.0)	1 (2.0)		
Incidence of adverse reactions	4 (8.0)	3 (6.0)	0.154	0.695

## 4 Discussion

Imaging findings from the present study demonstrated a significant improvement in both the Cobb angle and SVA of patients in both groups immediately postoperatively, as compared to their preoperative measurements, but the correction rate of the L group was higher immediately after operation, and the Cobb angle loss rate 12 months after operation was significantly lower than that of the S group, and the excellent rate of sagittal balance and the poor rate of vertebral body alignment were significantly better than those of the S group (*P* < 0.05). The core mechanism of this result lies in the load dispersion effect at the biomechanical level. LSF extends the fixation range to the spinal mechanical stability zone by spanning more than two intact vertebral bodies adjacent to the upper and lower segments of the deformity area, effectively dispersing the stress concentration at the two extremities of the fixed segment, avoiding the mechanical imbalance caused by “isolated fixation of deformity segments” during SSF (17). Previous studies have shown that long segment fusion can more effectively maintain the coronal and sagittal spinal balance, while conferring superior long-term stability in the correction of adult degenerative scoliosis (22). In addition, finite element analysis further confirmed that LSF can significantly reduce the peak stress of the fixed segment and adjacent segments, reduce the risk of vertebral body displacement and angulation, and provide a solid biomechanical foundation for the long-term maintenance of orthopedic effect (23). Specifically, long segment fusion improves the stiffness and stability of the overall internal fixation system by increasing the number of fixed segments, so as to better resist the non physiological activities of the spine under the postoperative dynamic load, and reduce the risk of internal fixation loosening, fracture or bone fusion failure caused by micro motion (22). In contrast, although short segment fusion has shorter operation time and less blood loss, it is difficult to fully correct serious structural deformities due to its limited fixation range, and it is more prone to the loss of Cobb angle and the progression of sagittal imbalance in long-term follow-up, which may be related to the increase of compensatory load of adjacent segments (24). Therefore, for patients with more complex adult degenerative scoliosis such as Lenke-Silva VI type, long segment fusion combined with osteotomy technology has clear advantages in obtaining and maintaining a good spinal force line, which is helpful to improve the clinical function prognosis and quality of life of patients, although the surgical trauma is relatively large (25). In addition, modern spine surgery increasingly emphasizes accurate surgical planning based on individualized anatomical parameters (such as pelvic incidence angle PI, sacral inclination angle SS, lumbar lordosis LL, etc.) to achieve optimal reconstruction of sagittal balance (23). The long segment fusion strategy is more conducive to the overall orthopedic design according to these parameters, ensuring the postoperative LL-PI matching, thus reducing the incidence of long-term adjacent segment disease (26, 27). It should be noted that the sagittal balance assessment in this study was restricted to SVA, without incorporating pelvic parameters such as PI, PT, and LL. Pelvic parameters are important for evaluating sagittal spinal-pelvic harmony, but the core focus of this study was the direct effect of fixation segment length on kyphotic deformity correction and global sagittal alignment restoration. SVA, as a widely recognized global sagittal balance indicator (17, 22), can effectively reflect the overall spinal force line improvement after osteotomy, which is sufficient to address the research objectives. However, the lack of pelvic parameter analysis may limit the comprehensive evaluation of sagittal spinal-pelvic coordination, which is a consideration for future studies. In conclusion, long segment fusion shows superior biomechanical stability and long-term orthopedic effect in the treatment of complex adult spinal deformities, and its clinical decision should comprehensively consider the severity of deformity, bone condition, age and general condition of patients.

Perioperative outcomes indicated that both the operative duration and intraoperative blood loss in Group S were significantly reduced compared with those in Group L (*P* < 0.05), which aligns with the research findings reported by Ma et al. (28) on thoracolumbar fractures. SSF achieved the advantage of “minimally invasive” by reducing the scope of soft tissue dissection, shortening the time of screw placement, and simplifying the intraoperative fluoroscopy steps. According to the concept of accelerated rehabilitation surgery (ERAS), trauma reduction can reduce the peak of postoperative inflammatory response (lower levels of C-reactive protein and interleukin-6), help reduce the systemic stress response, and thus promote early ambulation and functional recovery (29, 30). In this study, although group L experienced greater surgical trauma, the functional scores (ODI and VAS scores) 12 months after surgery were significantly better (*P* < 0.05), suggesting that patients’ tolerance to short-term trauma may exchange for better long-term spinal stability and functional benefits (22). Notably, no statistically significant difference was observed in the duration of hospital stay between the two groups (*P* > 0.05), indicating that LSF did not prolong the overall rehabilitation cycle, which may be related to better immediate postoperative stability, better pain control and lower complication rate in group L (31, 32). In addition, multimodal analgesic strategies under the ERAS pathway, especially opioid sparing regimens, have been shown to effectively manage postoperative pain while reducing adverse reactions, providing similar comfort base for patients with different fixation methods (33). Therefore, the results suggest that when making decisions on the treatment of spinal fractures, clinicians should not only focus on perioperative trauma indicators such as operation time and blood loss, but should comprehensively evaluate the individual situation, fracture type, expected stability needs and long-term functional prognosis of patients, in order to achieve individualized precise treatment in the true sense (29).

Complications are the key factors affecting the prognosis of kyphosis surgery, and early radiographic PJK and internal fixation loosening/fracture are the most common adverse events after surgery. Combined with previous studies and basic medical mechanisms, the occurrence of complications such as early radiographic PJK and loosening of internal fixation may be closely related to the disorder of tissue repair caused by local excessive inflammation after surgery, in addition to the difference in mechanical stability (34). Findings from the present study revealed that the incidence of early radiographic PJK, internal fixation loosening/fracture, and total complications in Group L was significantly lower than that in Group S (*P* < 0.05), suggesting that LSF has significant advantages in the prevention and control of complications. This result is different from some previous studies. For example, the study of Jiang et al (8) on adult patients with degenerative scoliosis believed that the perioperative complication rate of LSF was higher, but the PSM method was not used to control the baseline confounders in this study. Potential significant disparities in key indicators—including age, BMD, and deformity severity—between the two groups might have introduced bias into the results. Following PSM, the baseline characteristics of the two groups were balanced, which eliminated the interference of confounding factors and rendered the results more credible. A notable finding in our study was the lower complication rate in the LSF group compared to the SSF group, which contrasts with some historical cohorts reporting higher perioperative risks associated with longer constructs. This discrepancy is likely attributable to the PSM design of our study. In non-matched retrospective series, patients undergoing LSF often present with more severe deformities, older age, or worse comorbidities (selection bias), inherently predisposing them to complications. By strictly matching baseline characteristics—particularly the preoperative Cobb angle and age—we minimized this bias. Consequently, our results suggest that when patient baseline conditions are equivalent, the superior biomechanical stability of LSF may actually reduce implant-related complications (e.g., screw loosening, rod breakage) compared to the biomechanically more vulnerable SSF, without significantly increasing perioperative systemic risks.

Multivariate logistic regression analysis revealed that SSF was an independent risk factor for increased postoperative Cobb’ angle loss rate and a key risk factor for early radiographic PJK. As the primary independent risk factor for postoperative orthopedic loss and early radiographic PJK, the essence of SSF is the stress concentration effect caused by insufficient mechanical stability. After spinal correction, a mechanical interface is formed between the fixed segment and the adjacent unfixed segment. The interface fixed by the short segment is too close to the deformity area to effectively disperse and buffer the residual corrective torque after surgery, resulting in the high concentration of stress in the adjacent upper vertebra and its upper intervertebral disc structure (35). LSF transfers the high stress mechanical interface from the fragile deformity correction apex region to the higher spinal segment with relatively normal anatomy by extending the fusion range (36). This region has better vertebral bone conditions and more uniform mechanical conduction, thus reducing the risk of borderline kyphosis (37). In addition, the multi screw anchor system with LSF can provide stronger anti rotation and anti shear ability, especially in the weight-bearing state of the spine, which can effectively resist the biomechanics leading to orthopedic loss (38).

Based on our findings, we propose a preliminary clinical decision framework for determining the fusion length in ADS. LSF is strongly indicated for patients with larger significant sagittal imbalance, or osteoporosis, where a long lever arm is crucial to prevent correction loss and mechanical failure. Conversely, SSF might be cautiously considered for a select subgroup of patients: those with flexible curves, mild deformity, good bone quality, and significant medical comorbidities that prohibit prolonged surgical time. In these specific cases, SSF offers a ‘less is more’ approach, provided that the patient and surgeon accept a potentially higher risk of correction loss in exchange for reduced surgical trauma. Finally, future research should assess the cost-effectiveness of these strategies. While LSF involves higher upfront costs due to more implants and longer operating room time, it may offer long-term economic benefits by reducing the rate of revision surgeries and late-stage complications compared to SSF.

## 5 Study limitations

This study is a retrospective clinical controlled study. Although strict inclusion and exclusion criteria and independent researcher data collection and analysis ensure objectivity, there is still inherent selection bias in retrospective studies. The study is a single center with limited sample size and concentrated patient background, which may reduce the universality of the results. The 12-month follow-up is insufficient to assess long-term outcomes such as PJK and ASD. These complications are often related to long-term spinal biomechanical changes and degenerative processes, and their incidence and severity are more likely to be accurately reflected in follow-up periods of ≥2 years. The 12-month follow-up was insufficient to evaluate the long-term efficacy and long-term complication risk after spinal orthopedic surgery, and the confounding factors such as postoperative rehabilitation compliance and bone graft material type were not included in the regression model. In the future, it is suggested to carry out a multi center prospective randomized controlled study with large samples, extend the follow-up time, expand the range of independent variables, explore the biomechanical mechanism combined with basic and clinical research, and improve the reliability of the results and clinical reference value.

## 6 Conclusion

This study confirmed that LSF was significantly better than SSF in maintaining kyphosis, restoring sagittal balance, reducing early radiographic PJK, and internal fixation failure, but the operation time and blood loss increased; SSF has less trauma, but the risk of orthopedic loss is higher in older patients with osteoporosis and severe deformities. Regression analysis showed that SSF, preoperative Cobb ≥ 60°, and low BMD were independent risk factors for postoperative orthopedic loss, while SSF, low BMD, and advanced age were predictors of early radiographic PJK. Individualized decision-making is needed in clinic: long segment or bone cement reinforcement is preferred for patients with poor bone mass and severe deformity; In young patients with good bone mass, the short segment can be carefully selected and the follow-up can be strengthened. In the future, it is necessary to carry out prospective research, extend follow-up, explore AI aided decision-making system, and promote the development of orthopedic treatment precision and value medicine.

## Data Availability

The original contributions presented in the study are included in the article/Supplementary Material, further inquiries can be directed to the corresponding author.
